# The Feed and Sleep method: how to perform a cardiac MRI in the 1st year of life without the need for General Anesthesia

**DOI:** 10.1186/1532-429X-13-S1-P224

**Published:** 2011-02-02

**Authors:** Jonathan D Windram, Lars Grosse-Wortmann, Masoud Shariat, Mary-Louise Greer, Shi-Joon Yoo

**Affiliations:** 1 The Hospital for Sick Children, Toronto, ON, Canada

## Introduction

MRI in small children as a general rule necessitates the use of general anesthesia. We describe our initial results with a new technique which we name the feed and sleep method whereby an infant can undergo a Cardiac MRI without the need for general anesthesia or sedation.

## Methods

The infant is starved for 4 hours prior to the scan and is then fed by his mother prior to the scan. He is then swaddled with 1 to 2 infant sheets before placed within a vacuum-bag immobilizer. As air is removed from the bag, the immobilizer becomes a rigid cradle that fits the infant’s body. We prioritize the sequences to target the area of importance first according to the purposes of the study and in the order of clinical importance.

## Results

Between Jan and July 2010 a total of 12 infants median age 14 days (minimum 2 days maximum 150 days ) have undergone CMR studies via this method. All were performed successfully with no distress to the infant. The average scan time was 45.5 min (minimum 24, maximum 65 min). All had complex congenital heart defects (Table 1) and all planned sequences were able to be acquired ensuring the scans were of sufficient quality to allow accurate diagnosis and to plan appropriate future surgery.

**Table 1:** 

Pt	Diagnosis	Time of scan (min)	Age (days)
1	Right atrial isomerism, Atrioventricular Septal Defect with total anomalous pulmonary venous drainage	37	2
2	Left ventricular hypoplasia	48	12
3	Right atrial isomerism, Atrioventricular Septal Defect with total anomalous pulmonary venous drainage	65	60
4	Total anomalous pulmonary venous drainage	46	150
5	Hypoplastic Left heart syndrome, Blalock Taussig Shunt, Left pulmonary artery stenosis.	33	15
6	Arteriovenous malformation	57	14
7	Supracardiac total anomalous pulmonary venous drainage, left atrial isomerism with interrupted IVC	57	12
8	Tetralogy of Fallot	59	4
9	Aortic coarctation	24	30
10	Congenital Correction of Transposition of the Great Arteries, Atrial Septal Defect, Ventricular Septal Defect, central shunt	58	35
11	Double Outlet Right Ventricle, sub-pulmonary VSD, Interupted Aortic Arch (type A)	39	4
12	Absent right pulmonary artery	27	14

## Conclusion

Using this technique infants under the age of 6 months can complete a cardiovascular MRI, without the need for general anesthesia. We advocate the incorporation of this safe and reliable technique into routine clinical practice.

